# Predictors for the Recurrence of Clinically Uterine-Confined Endometrial Cancer and the Role of Cytokeratin Immunohistochemistry Stain in the Era of Sentinel Lymph Node Mapping

**DOI:** 10.3390/cancers14081973

**Published:** 2022-04-13

**Authors:** Wan-Hua Ting, Shu-Wei Hsieh, Hui-Hua Chen, Ming-Chow Wei, Ho-Hsiung Lin, Sheng-Mou Hsiao

**Affiliations:** 1 Department of Obstetrics and Gynecology, Far Eastern Memorial Hospital, New Taipei 220, Taiwan; stellatingwh@gmail.com (W.-H.T.); thandaaye24@gmail.com (H.-H.C.); wei@mail.femh.org.tw (M.-C.W.); hhlin@ntuh.gov.tw (H.-H.L.); 2 Department of Industrial Management, Asia Eastern University of Science and Technology, New Taipei 220, Taiwan; 3 Department of Anatomical Pathology, Far Eastern Memorial Hospital, New Taipei 220, Taiwan; shuweihsieh202bce@gmail.com; 4 Department of Obstetrics and Gynecology, National Taiwan University College of Medicine and National Taiwan University Hospital, Taipei 100, Taiwan; 5 Graduate School of Biotechnology and Bioengineering, Yuan Ze University, Taoyuan 32003, Taiwan

**Keywords:** sentinel lymph node, lymphadenectomy, ultrastaging, endometrial cancer, cytokeratin stain

## Abstract

**Simple Summary:**

Sentinel lymph node (SLN) mapping in women with endometrial cancer is gradually gaining popularity worldwide. The objectives of this retrospective study were to elucidate the predictors for cancer recurrence in the era of SLN mapping, and to compare the clinical outcomes between SLN mapping and traditional lymphadenectomy, as well as to investigate the role of cytokeratin immunohistochemistry stain in detecting lymph node metastases. Para-aortic lymph node metastasis was found to be the sole predictor for cancer recurrence. Cytokeratin immunohistochemistry stain detects more lymph node metastases. In addition, both SLN mapping and traditional lymphadenectomy have similar probabilities of cancer recurrence.

**Abstract:**

Background: The primary objective of this study was to elucidate the predictors for cancer recurrence in women with clinically uterine-confined endometrial cancer in the era of sentinel lymph node (SLN) mapping. Methods: All consecutive women with clinically determined uterine-confined endometrial cancer who had lymph node assessment by either SLN mapping or traditional pelvic lymphadenectomy were reviewed. Results: Women in the SLN mapping group had lower total dissected pelvic nodes, lower incidence of para-aortic lymph node dissection, less intraoperative blood loss and lower complication rates, but a longer operation time compared to the traditional lymphadenectomy group. Para-aortic lymph node metastasis (hazard ratio = 7.60, *p* = 0.03) was the sole independent predictor for recurrence-free survival. In addition, the utilization of cytokeratin immunohistochemistry stain detected more lymph node metastases (adjusted odds ratio = 3.04, *p* = 0.03). Recurrence-free survival did not differ between SLN mapping and traditional lymphadenectomy groups (*p* = 0.24). Conclusions: Para-aortic lymph node metastasis is an important predictor of cancer recurrence. Women with negative hematoxylin and eosin stain should undergo cytokeratin immunohistochemistry stain to increase the detection rate of positive lymph node metastasis. Besides, the probabilities of recurrence seem to be similar between SLN mapping and traditional lymphadenectomy groups in women with clinically uterine-confined endometrial cancer.

## 1. Introduction

Sentinel lymph node (SLN) mapping in women with endometrial cancer is gradually gaining popularity worldwide. Approximately 10% to 15% of women thought to have cancer confined to the uterus at initial diagnosis are found to have lymph node metastases. The association of lymph node metastases with disease outcome has ensconced lymph node status as an integral component to the staging of this disease [1]. The role of traditional lymphadenectomy remains debatable as it could possibly result in exposure of many women to unnecessary extensive surgical risk. There is emerging evidence supporting the utilization of SLN mapping in selected cases with low risk for nodal disease [2,3]. SLN mapping identified more than twice as many lymph node metastases with a near doubling of adjuvant therapy [4]. Lower pelvic side wall recurrence has been reported in the SLN group, but no significant impact was detected on overall disease recurrence between those with and without positive SLN [5,6]. A recent multi-institutional international retrospective study also highlighted similar survival outcomes regardless of the type of nodal assessment in high-risk endometrial cancer, suggesting the possibility of substituting traditional lymphadenectomy with SLN mapping [7]. In fact, the National Comprehensive Cancer Network (NCCN) guidelines have approved the execution of SLN mapping for endometrial cancer staging procedures for both low- and high-risk endometrial cancer, thus supporting the value of incorporating SLN mapping into clinical practice [8].

SLN mapping is almost invariably combined with additional pathologic assessment via serial sectioning with ultrastaging in order to discover low volume metastatic disease. SLN is initially examined using hematoxylin and eosin (H&E) stain. If the H&E assessment is negative, additional serial sectioning with review of multiple H&E-stained slides is done [9]. Multiple ultrastaging methods are adopted in different institutions, with differences in macroscopic slicing (bread-loaf/longitudinal), number of microscopic slides, and distance between slides [10]. Anti-cytokeratin AE1:AE3 is the most commonly used broad-spectrum cytokeratin antibody cocktail in pathological assessment [11]. An additional 4.5% of micrometastases were detected with immunohistochemistry (IHC) stain in dissected SLNs, which may have been otherwise missed by routine H&E stain [9].

Our institution started to adopt the SLN mapping technique in women with clinically uterine-confined endometrial cancer from July 2017. We were interested in the impact of the SLN mapping technique and histopathologic examination methods on the clinical outcomes of women with clinically uterine-confined endometrial cancer. Thus, the primary objective of this study was to elucidate the predictors for cancer recurrence in women with clinically uterine-confined endometrial cancer in the era of SLN mapping. The secondary objectives were to compare the clinical outcomes between SLN mapping and traditional lymphadenectomy groups, and to investigate the role of cytokeratin IHC stain in detecting lymph node metastases.

## 2. Materials and Methods

This retrospective study analyzed all consecutive women aged 20 and above with documented endometrial cancer of any histology on pathology specimens from either endometrial sampling or dilation and curettage, who received staging operations in a tertiary referral center from January 2008 till May 2021. Only women with clinically determined uterine-confined disease based on preoperative physical examination and radiological survey, including chest X-ray film, computerized tomography and magnetic resonance imaging, were included. Women were staged according to the 2009 International Federation of Gynecology and Obstetrics (FIGO) staging system. This study was approved by the Research Ethics Review Committee of the hospital.

**Figure 1:**
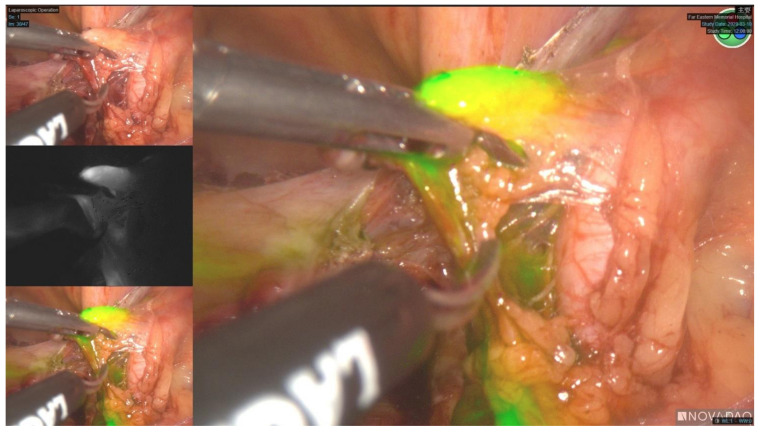
Sentinel lymph node mapping over right hemipelvis.

All women underwent staging surgery either via laparotomic, laparoscopic, or robotic approach. The choice of surgical method was at the surgeon’s discretion and personal financial consideration. The standard staging procedures included total hysterectomy, bilateral salpingo-oophorectomy, lymph node assessment and dissection, biopsy of any suspicious lesions, and peritoneal or ascites cytology. Omental biopsy was performed in those with serous carcinoma, clear cell carcinoma, or carcinosarcoma histologies. SLN mapping and/or robotic surgery were performed on women who could afford the additional medical cost incurred by the application of the endoscopic fluorescence imaging system (PINPOINT, Novadaq, Mississauga, ON, Canada) for SLN mapping, and the da Vinci Si Surgical System (Intuitive Surgical, Inc., Sunnyvale, CA, USA), since these costs are not covered by the National Health Insurance in Taiwan. Para-aortic lymphadenectomy was done at the discretion of the attending surgeon based on the presence of risk factors such as high-risk histologies, suspicious nodal status, deep myometrial invasion, the presence of extra-uterine disease, or the presence of positive para-aortic SLN mapping. In the SLN group, indocyanine green (ICG) tracer was diluted to a dose of 2.5 mg/mL and injected into the cervix at 3 o’clock and 9 o’clock positions. At each position, 1 mL of ICG tracer was injected to cervical submucosa (1 cm in depth) and deep cervical stroma (3 cm in depth) respectively, achieving a total volume of 4 mL. Detection of SLN was accomplished by visualization of colored lymph nodes via the endoscopic fluorescence imaging system (Figure 1). The protocol for SLN mapping in our institution was adopted from the SLN algorithm developed by the Memorial Sloan Kettering Cancer Center [12]. All mapped SLNs and grossly enlarged suspicious nodes, regardless of mapping, were removed. A side specific pelvic lymphadenectomy (including external iliac, internal iliac and obturator region) was performed if mapping failed on a hemipelvis [12].

SLN specimens were processed according to the size of the nodes. If the node was small (i.e., equal or less than 1 cm in its long axis), it was bivalved and examined by pathologists under routine H&E stain. If the node was large (i.e., more than 1 cm), it was sliced at 2–3 mm intervals perpendicular to the long axis. Additional cytokeratin IHC stain was performed at the discretion of the individual pathologist and/or if routine H&E stain was negative. Nodal status was reported in a standardized fashion according to the American Joint Committee on Cancer [13]. Macrometastasis was defined as foci of metastasis greater than 2 mm; micrometastasis was defined as disease volume 0.2–2 mm; and isolated tumor cells were defined as foci of disease measuring less than 0.2 mm in its greatest dimension [13].

Disease recurrence was assessed according to the appearance of abnormal radiological findings, or histological proof from biopsy analyses, whichever occurred first. Recurrence-free survival (RFS) was defined as the time interval from the date of surgery to clinically defined recurrence, disease progression, or the last follow-up. Endometrial cancer-specific survival was measured from the date of surgery to the date of death related to endometrial cancer progression, or the last follow-up. Overall survival was measured from the date of surgery to the date of death, or the last follow-up.

Stata version 11.0 (Stata Corp, College Station, TX, USA) was used for statistical analyses. The Chi-square test and Fisher’s exact test were used as appropriate. A *p*-value less than 0.05 was considered statistically significant. Survival curves were generated using the Kaplan–Meier method, and the statistical differences in the survival curves were estimated with the log-rank test. A Cox proportional hazards model was used to identify the predictors of RFS. A multivariable backward stepwise Cox proportional hazard model using all variables with *p* < 0.05 in the univariate analysis was performed till all the remaining variable(s) were significant. In addition, logistic regression analysis was used to predict the presence of lymph node metastasis. A multivariable Cox proportional hazards model was performed by using clinically interested variables and all variables with *p* < 0.05.

## 3. Results

A total of 334 women with clinically uterine-confined endometrial cancer were included in the study. The clinico-pathological characteristics of these women are shown in Table 1. Sixty-two (18.6%) women had SLN mapping, whereas the remaining (*n* = 272) received traditional lymphadenectomy. Except body mass index (BMI), operative method and tumor size, baseline characteristics were similar in both groups (Table 1).

Women in the SLN mapping group had lower total dissected pelvic nodes, lower incidence of para-aortic lymph node dissection, less intraoperative blood loss, lower complication rates, and shorter median follow-up time but a longer operation time compared to the traditional lymphadenectomy group (Table 1).

**Figure 2:**
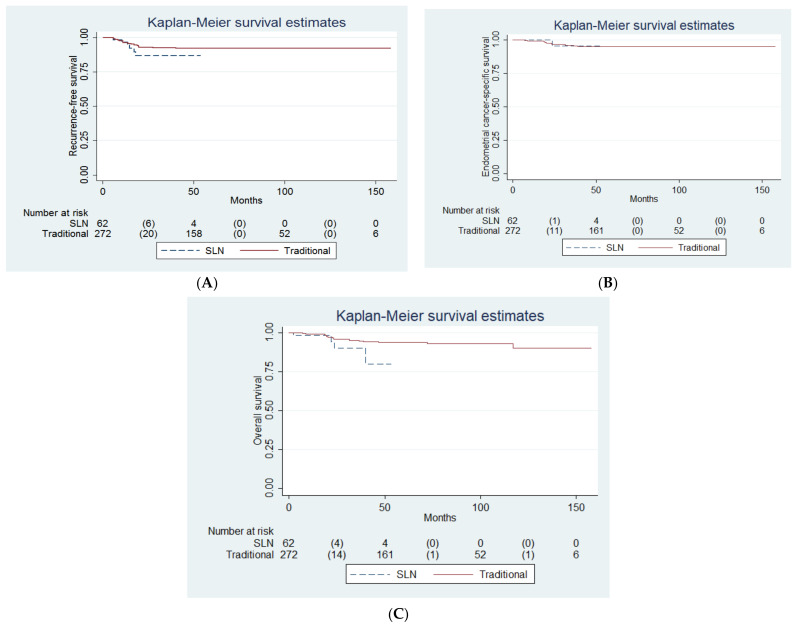
Probabilities of (**A**) recurrence-free survival, (**B**) endometrial cancer-specific survival, and (**C**) overall survival between the traditional lymphadenectomy and sentinel lymph node mapping groups in women with clinically uterine-confined endometrial cancer (*n* = 334).

Survival analysis of both groups did not reveal any difference in RFS, endometrial cancer-specific survival, and overall survival (Figure 2A–C and Table 1).

The location of detected SLNs in women who underwent SLN mapping is shown in Table 2. The most common location of detected SLNs was the external iliac area, followed by the obturator area (Table 2).

The univariate Cox proportional hazard model revealed that age, BMI, endometrioid cell, cell grade, deep myometrial invasion, lymphovascular space invasion, tumor size, pelvic and para-aortic lymph node metastases, and stage were predictors for RFS. Nonetheless, the presence of para-aortic lymph node metastasis was the sole independent predictor of RFS in the multivariable Cox proportional hazard model (hazard ratio = 7.60, *p* = 0.03, Table 3).

The survival rates at 48 months after surgery were 92.0% and 86.7% in the traditional lymphadenectomy and SLN mapping groups, respectively. The hazard ratio for the utilization of the SLN mapping technique was 1.73 (*p* = 0.24) in the univariate analysis (Table 3). Thus, the statistical power to discriminate between-group differences of RFS in women who underwent traditional lymphadenectomy or SLN mapping technique was estimated to be 0.39.

**Figure 3:**
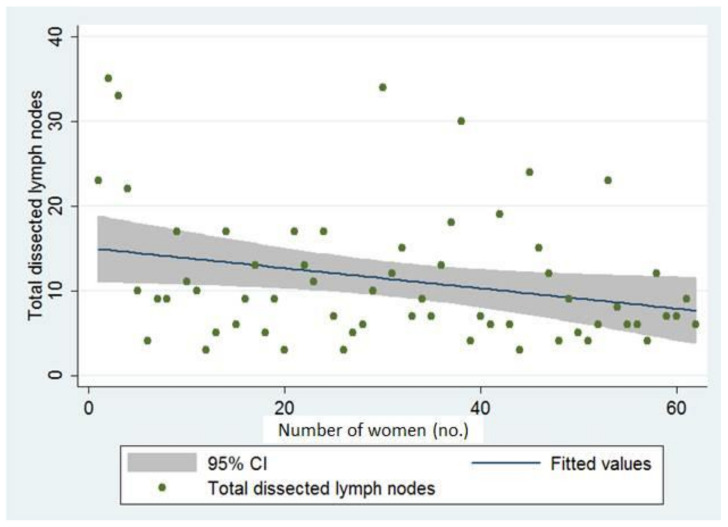
Total number of dissected pelvic lymph nodes versus the number of women across time in the sentinel lymph node group (*n* = 62).

The total number of dissected pelvic lymph nodes versus the number of women in the SLN mapping group is shown in Figure 3. In most cases, the total number of dissected pelvic lymph nodes was under 20 (Figure 3).

In this cohort, 24 cases in the SLN mapping group (*n* = 62) and 22 cases in the lymphadenectomy group (*n* = 272) had additional cytokeratin IHC staining (*p* = 0.02, Fisher’s exact test). Lymph node metastases were found in 7 women of the cytokeratin IHC staining group and 18 women in the non-cytokeratin IHC staining group. A significantly higher proportion of positive lymph node metastasis was identified using cytokeratin IHC stain, compared to those without (7/46 vs. 18/288, *p* = 0.03, chi-square test).

Univariate logistic analysis revealed that the use of cytokeratin IHC stain was a significant predictor for detecting lymph node metastasis (odds ratio = 2.69, *p* = 0.04, Table 4). Owing to a higher proportion of women who received cytokeratin IHC staining in the SLN group compared to the traditional group (24/62 vs. 22/272, *p* < 0.001, Fisher’s exact test), we adjusted the variable SLN mapping, and the use of cytokeratin IHC stain remained a significant predictor for positive lymph node metastasis (odds ratio = 3.04, *p* = 0.03, Table 4).

## 4. Discussion

In this study, we found the presence of para-aortic lymph node metastasis as the sole independent predictor of RFS in the multivariable analysis (Table 3), compatible with the study result of a retrospective study by Chen et al. (hazard ratio = 11.11, *p* < 0.001) [14]. Similarly, lymph node status is considered to be the most important predictor for cancer outcome in a review article by Pijnenborg et al. [15]. It is worth mentioning that combined chemotherapy and external beam radiotherapy had a superior survival rate for women with stage IIIC2 endometrioid endometrial cancer, compared to single therapy alone [16]. However, the prognosis of women with stage IIIC2 non-endometrioid endometrial cancer remained poor regardless of the adjuvant therapy administered [16].

A significantly higher proportion of positive lymph node metastasis was identified by using cytokeratin IHC staining, compared to those without (7/46 vs. 18/288, *p* = 0.03, chi-square test) in our study. The use of cytokeratin IHC stain was also a predictor for detecting lymph node metastasis in the multivariate analysis (odds ratio = 3.04, *p* = 0.03, Table 4). Cytokeratins are a group of intermediate filament proteins that are essential tissue-specific constituents of both normal and malignant epithelial cells, and the cytokeratin patterns of the individual epithelia are generally well preserved even in their derivative carcinomas and metastases. Normal lymph node tissue does not contain cytokeratins, therefore they are not usually found in any type of cell constituting lymph nodes except during development of a tumor metastasis or inflammation [17]. Cytokeratin IHC staining using anti-cytokeratin AE1:AE3 helps in the identification of occult metastasis that would be difficult to detect by H&E, including micrometastasis and isolated tumor cells [18,19]. Thus, based on our study results, we recommend the integration of cytokeratin IHC staining of SLNs for women with negative H&E stain.

Our study did not demonstrate a survival difference in women with clinically uterine-confined endometrial cancer who underwent SLN mapping or traditional lymphadenectomy, despite its limited statistical power (i.e., the power to discriminate the between-group difference in RFS was 0.39). Several studies have reported that SLN mapping has at least acceptable diagnostic accuracy. Ducie et al. reported that the SLN mapping algorithm with ultrastaging histopathologic method provides similar detection rates of stage IIIC endometrial cancer compared with traditional lymphadenectomy [20]. In the FIRES study, women with clinical stage I endometrial cancer underwent SLN mapping, followed by complete lymphadenectomy. The FIRES study found that SLN mapping had a high degree of diagnostic accuracy in detecting metastasis, but SLN biopsy did not identify metastasis in 3% of women with node-positive disease [21]. In the SENTOR study, women with clinical stage I, grade 2, and high-grade endometrial cancer underwent SLN mapping, followed by complete lymphadenectomy. Similarly, the SENTOR study found that SLN mapping had acceptable diagnostic accuracy in detecting metastasis for women with high grade endometrial cancer [22]. Most of the comparative studies included bilateral pelvic lymphadenectomy as a reference standard in the SLN mapping group. In our study, no additional back-up lymphadenectomy was performed in the SLN mapping group unless due to failure of mapping or the presence of enlarged nodes. Thus, the finding of similar RFS between the traditional lymphadenectomy and SLN mapping groups in our study might provide a supporting rationale to perform SLN mapping and biopsy without additional back-up lymphadenectomy in clinically uterine-confined endometrial cancer patients (Figure 2A).

In our study, the rate of all complications (including lymphedema and lymphocyst) was higher in the traditional lymphadenectomy group compared to the SLN mapping group (27% vs. 13%, *p* = 0.02, Table 1). It is, however, worthwhile to emphasize that the number of women in the traditional lymphadenectomy group is four times more than the SLN mapping group in this study. Traditional lymphadenectomy is often associated with higher risk of extremity lymphedema and lymphocysts and vascular injury [23]. The estimated incidence of lymphedema after complete lymphadenectomy is 20% to 30% [24]. Abu-Rustum, et al., showed that removal of 10 or more regional lymph nodes possessed a higher risk for developing symptomatic leg lymphedema [25]. SLN mapping was reported to have fewer complications, such as lymphedema and lymphocyst. The lower complication rate in the SLN mapping group as shown in our study seems to be in line with the reported findings [25].

A recent study showed that the median size of the lymphocyst was larger and more likely to be symptomatic in the laparotomy group than the laparoscopic group [26]. In our study, the size of the lymphocyst had a trend to be significantly larger in the laparotomy group than the laparoscopic/robotic group (4.9 ± 1.8 cm vs. 4.1 ± 3.3 cm, *p* = 0.059). In addition, if we utilized the use of laparotomy and the use of lymph node mapping to predict the presence of lymphocyst/lymphedema, we did find that the use of laparotomy (odds ratio = 3.30, 95% confidence interval = 1.32 to 8.27, *p* = 0.01) was an independent factor to predict the presence of lymphocyst/lymphedema, but not the use of SLN mapping (odds ratio = 0.69, 95% confidence interval = 0.18 to 2.55, *p* = 0.58). Thus, the higher percentage of lymphocyst/lymphedema in the traditional group in this study might be partly related to a higher percentage of laparotomy in the traditional group.

In our institute, large (i.e., >1 cm) lymph nodes were sliced at 2–3 mm intervals perpendicular to the long axis, and additional cytokeratin IHC staining was performed for lymph nodes with negative H&E stain. Ultrastaging involves additional sectioning and staining of the lymph nodes in order to examine the lymph nodes for low volume metastatic disease. There is no universal consensus on a standardized well-defined ultrastaging protocol for SLN evaluation in gynecologic malignancies [10]. A systematic review showed higher detection rate of SLN metastases using the ultrastaging method but failed to show a positive correlation between the number of ultrastaging slides and detection rate [10]. In addition, Kim, et al., also suggested ultrastaging may be omitted in early stage endometrial cancer without any myoinvasion [9]. Ultrastaging for all dissected lymph nodes is a labor intensive and time-consuming work, and indubitably increases cost. Current practice in Memorial Sloan Kettering Cancer Center is to forgo ultrastaging in women with non-myoinvasive low-grade endometrioid cancer owing to the low incidence of isolated tumor cells and micrometastasis in these women, and for the sake of time and cost effectiveness [27]. With no survival difference between the two groups of women in our study (*p* = 0.24, Figure 2 and Table 2), the adoption of the SLN mapping technique, combined with the histopathologic slicing method of SLNs and the use of cytokeratin IHC staining in our institute, seems to be a feasible option for women with clinically uterine-confined endometrial cancer.

Longer operative time and lesser surgical blood loss were seen in the SLN mapping group compared to the traditional lymphadenectomy group (Table 1). After adjusting the confounding factor (surgical method), the use of the SLN mapping technique (coefficient = 46.0 min, *p* = 0.002) remained a predictor for operation time. During SLN mapping, the retroperitoneal space (such as paravesical space and obturator fossa) should be carefully dissected to minimize vascular bleeding for optimal visualization of the mapped lymph node(s) in a bloodless space. Besides, our surgeons were at only the half-way stage of the learning curve when this retrospective study was done. These two points might explain the longer operative time in the SLN mapping group. In both LAP2 and LACE trials, longer operative time was also seen in the laparoscopic group, but this did not translate into higher postoperative surgical complications, thus supporting the non-inferiority of minimally invasive approaches in women with early-stage endometrial cancer [28,29].

Changes in available technology have expanded the interest in creating personalized medicine platforms to allow tailoring of medical treatment to the individual characteristics of each patient. In particular, for elderly oncology women, the role of the minimally invasive procedure has an even more valid meaning [30]. In our study, almost a quarter of women (35/147) in the minimally invasive group were aged 65 and over, none of whom were converted to laparotomy. Age is therefore not a contraindication for the minimally invasive approach. Besides, minimally invasive salvage lymphadenectomy has been shown to provide a comparable oncological outcome in selected women with localized nodal recurrence [31]. Technological evolution and innovation have made personalized therapy a reality, and therefore should be incorporated in the multimodal therapeutic approach of oncologic women.

The most common location of SLN in this study was the external iliac region, followed by the obturator region (Table 2). These observations are compatible with other published studies [21,32]. How, et al., demonstrated the capability of SLN mapping to map out areas not typically included in standard lymphadenectomy, and this could play a beneficial role in subsequent treatment [32]. As shown in our study, para-aortic lymph node metastasis was the sole predictor for RFS in the multivariable analysis (Table 3). We did detect eight cases with para-aortic SLNs in our study. It was found that cervical injection yielded a low para-aortic SLN detection rate, and thus possibly missed a small proportion of women who would have isolated positive para-aortic nodes with negative pelvic nodes [21,33,34]. However, the risk of isolated para-aortic metastases in early clinical stage endometrial cancer is rare (approximately 1%) even in high-grade lesions [35]. It is recommended to include para-aortic lymphadenectomy in cases with grossly enlarged lymph nodes suspected to be malignant, and selective high-risk situations such as high tumor volume, high histology grade and deep myometrial invasion [36,37,38].

The main concerns regarding SLN mapping include technical proficiency and false negatives attributable to failure to detect SLNs [39]. Khoury-Collado, et al., suggested that a learning curve of 30 cases is required to achieve SLN detection rates of >90% [40]. Another study by Tucker, et al., reported a 5% and an 11% increase in the odds of successful SLN mapping and removal of lymph node-containing tissue, respectively, with each additional procedure performed [41]. In our study, the SLN detection rate of at least one hemipelvis was 94%; successful mapping on at least one hemipelvis and bilateral hemipelvis mapping were 26% and 68%, respectively (Table 1). The issue of false negatives can be overcome by the application of the Memorial Sloan Kettering Cancer Center SLN algorithm, which includes ipsilateral hemipelvic lymph node dissection in women in whom no SLN is detected, with removal of all suspicious nodes irrespective of SLN mapping findings [6].

The strength of this study is its large sample size. However, we acknowledge that the clinical evidence of this study is limited due to its retrospective, limited sample size of SLN mapping group; limited cases with cytokeratin IHC staining; and nonrandomized nature. In addition, we used a modified ultrastaging method to examine the dissected lymph nodes. Furthermore, this study covered a span of almost 14 years, and SLN mapping has only been widely used since 2021 in our institution, leading to a significant difference in the median follow-up time between the two groups, which therefore possibly led to outcome bias. The common adjuvant chemotherapy regimen for high-risk groups (i.e., platinum plus doxorubicin or platinum plus paclitaxel) did not change during the 14-year period in our institution. However, differences in surgical techniques might have occurred over that period. Moreover, this study includes cases performed by all gynecologic surgeons in our institute, thus the differences in clinical-surgical competency of these surgeons might lead to outcome bias.

## 5. Conclusions

Para-aortic lymph node metastasis is an important predictor of cancer recurrence. Women with negative H&E stain should undergo cytokeratin IHC stain to increase the detection rate of positive lymph node metastasis. In addition, the probabilities of recurrence seem to be similar between the SLN mapping and traditional lymphadenectomy groups in women with clinically uterine-confined endometrial cancer.

## Figures and Tables

**Table 1:** Comparisons of the baseline data and clinical outcomes between the traditional lymphadenectomy and sentinel lymph node mapping groups in women with clinically uterine-confined endometrial cancer (*n* = 334).

Variables	Traditional (*n* = 272)	SLN (*n* = 62)	† *p*
Age (years)	55.5 ± 10.0	57.3 ± 10.6	0.08
Body mass index (kg/m^2^)	27.0 ± 6.1	28.1 ± 4.4	0.04
Operative method			
Laparotomic staging	178 (65)	8 (13)	<0.001
Laparoscopic staging	61 (22)	44 (71)	
Robotic staging	33 (12)	9 (15)	
ECOG score			
0	122 (45)	22 (35)	0.41
1	141 (52)	39 (63)	
2	5 (2)	1 (0)	
3	3 (1)	0 (0)	
Parity	2.1 ± 1.3	1.8 ± 1.3	0.23
Endometrioid cell type	229 (84)	52 (84)	0.70
Cell grade			
1	125 (46)	31 (50)	0.21
2	91 (33)	14 (23)	
3	42 (15)	15 (14)	
Deep (>1/2) myometrial invasion	64 (24)	15 (24)	0.99
Lymphovascular space invasion	97 (36)	21 (34)	0.26
Lymph node pelvic metastasis	18 (7)	5 (8)	0.69
Tumor size (cm)	3.2 ± 2.6	2.4 ± 1.6	0.04
Washing cytology			
Malignant cell	10 (4)	2 (3)	0.20
Atypical cell	53 (19)	19 (31)	-
CA-125 (U/mL)	61.6 ± 142.4	47.3 ± 142.4	0.59
SLN mapping			
Left hemipelvis	-	51 (82)	-
Right hemipelvis	-	49 (79)	-
Bilateral hemipelvis	-	42 (68)	-
Unilateral hemipelvis	-	16 (26)	-
Mapping failure	-	4 (6)	-
Total number of dissected lymph nodes	18.0 ± 9.8	11.3 ± 7.9	<0.0001
Total number of positive pelvic lymph nodes	0.19 ± 0.97	0.18 ± 1.15	0.48
Para-aortic lymph nodes dissection	54 (20)	8 (13)	<0.001
Stage			0.71
IA	187 (69)	39 (63)	
IB	46 (17)	13 (21)	
II	13 (5)	2 (3)	
IIIA	6 (2)	2 (3)	
IIIC1	15 (6)	4 (6)	
IIIC2	4 (1)	1 (2)	
IVB	1 (0)	1 (2)	
Adjuvant radiotherapy	135 (48)	27 (44)	0.47
Adjuvant chemotherapy	48 (11)	12 (19)	0.41
Operation time (min)	185 ± 72	209 ± 70	0.008
Blood loss (mL)	349 ± 300	196 ± 197	<0.0001
Complications	74 (27)	8 (13)	0.02
Lymphocyst/lymphedema	33 (7)	3 (5)	0.11
Average size of lymphocyst (cm)	4.9 ± 2.2	2.5 ± 0.8	0.03
Median follow-up (months)	65.0 ± 39.9	22.6 ± 12.8	<0.0001
Recurrence	20 (11)	6 (10)	‡ 0.24
Death	16 (6)	4 (7)	‡ 0.09
Endometrial cancer related	11 (4)	1 (2)	‡ 0.79
Other causes (i.e., sepsis, *n* = 4; second malignancy, *n* = 2; pneumonia, *n* = 2)	5 (2)	3 (5)	-

Values are expressed as mean ± standard deviation or number (percentage). ECOG = Eastern Cooperative Oncology Group; SLN = sentinel lymph node mapping. † Wilcoxon rank-sum test, chi-square test or Fisher’s exact test. ‡ Log-rank test.

**Table 2:** Location of detected sentinel lymph nodes in women who underwent sentinel lymph node mapping (*n* = 62).

Location	Left Hemipelvis (*n* = 51)	Right Hemipelvis (*n* = 49)
External iliac	38 (75)	34 (69)
Obturator	24 (47)	26 (53)
Common iliac	2 (4)	3 (6)
Presacral	0 (0)	1 (2)
Para-aortic	0 (0)	2 (4)
Not specified	5 (10)	6 (12)

Values are expressed as number (percentage).

**Table 3:** Predictors for recurrence-free survival in women with clinically uterine confined endometrial cancer (*n* = 334).

Variables	Univariate		Multivariable	
	Hazard Ratio (95% CI)	† *p*	Hazard Ratio (95% CI)	‡ *p*
SLN mapping	1.73 (0.69, 4.34)	0.24	3.54 (0.52, 23.86)	0.19
Age (years)	1.05 (1.01, 1.08)	0.02	1.00 (0.94, 1.07)	0.97
BMI (kg/m^2^)	0.93 (0.87, 0.99)	0.02	0.88 (0.74, 1.05)	0.15
ECOG scale				
0 (reference)	1.00	-	-	-
1	0.71 (0.33, 1.57)	0.40	-	-
2	1.85 (0.24, 14.1)	0.55	-	-
3	3.80 × 10^−15^ (0, infinity)	1.00	-	-
Parity	1.20 (0.84, 1.73)	0.31	-	-
CA-125 (U/mL)	1.00 (1.00, 1.00)	0.91	-	-
Surgical method				
Laparotomic (reference)	1.00	-	-	-
Laparoscopic	1.15 (0.48, 2.75)	0.75	-	-
Robotic	1.37 (0.45, 4.15)	0.58	-	-
Total number of dissected pelvic lymph node	1.00 (0.96, 1.04)	0.89	-	-
Endometrioid cell type	0.17 (0.08, 0.36)	<0.001	0.84 (0.17, 4.10)	0.83
Cell grade				
1 (reference)	1.00	-	1.00	-
2	7.90 (1.73, 36.1)	0.008	3.57 (0.37, 34.85)	0.27
3	19.9 (4.46, 89.2)	<0.001	7.13 (0.63, 80.85)	0.11
Deep (>1/2) myometrial invasion	2.59 (1.18, 5.71)	0.02	0.47 (0.10, 2.33)	0.36
LVSI	4.75 (1.89, 11.98)	0.001	0.75 (0.16, 3.55)	0.72
Tumor size (cm)	1.01 (1.01,1.02)	<0.001	1.01 (0.99, 1.04)	0.28
Pelvic lymph node metastasis	6.90 (3.00, 15.88)	<0.001	1.52 (0.29, 7.95)	0.62
Para-aortic lymph node metastasis	12.74 (3.90, 41.59)	<0.001	7.60 (1.28, 45.16)	0.03
Ascites cytology.				
Normal cell	1.00	-	-	-
Atypical cell	1.79 (0.76,4.21)	0.19	-	-
Malignant cell	2.51 (0.57, 11.00)	0.22	-	-
Stage				
IA	1.00 (reference)	-		
IB	2.56 (0.99, 6.59)	0.052		
II	1.70 × 10^−15^ (0, infinity)	1.00		
IIIA	1.71 × 10^−15^ (0, infinity)	1.00		
IIIC1	4.77 (1.52, 14.97)	0.008		
IIIC2	14.6 (4.05, 52.79)	<0.001		
IVB	22.76 (2.79, 185.42)	0.003		

BMI = body mass index; CI = confidence interval; ECOG = Eastern Cooperative Oncology Group; LVSI = lymphovascular space invasion; SLN = sentinel lymph node mapping. † Univariate Cox proportional hazards model. ‡ Multivariable Cox proportional hazards model by using clinically interested variables and all variables with *p* < 0.05. The variable “stage” was excluded in the multivariable analysis owing to its significant correlation with positive para-aortic lymph node metastasis (Spearman’s rho = 0.38, *p* < 0.001.). In addition, the use of sentinel lymph node mapping was included in this model owing to clinical interest.

**Table 4:** Predictors of lymph node metastasis.

Variables	Univariate		Multivariate	
	**Odds Ratio (95% CI)**	**† *p***	**Odds Ratio (95% CI)**	**‡ *p***
SLN mapping	1.11 (0.40, 3.07)	0.85	0.73 (0.24, 2.24)	0.58
Cytokeratin IHC staining	2.69 (1.06, 6.86)	0.04	3.04 (1.09, 8.44)	0.03

CI = confidence interval; IHC = immunohistochemistry; SLN = sentinel lymph node. † Univariate logistic regression analysis. ‡ Multivariable logistic regression analysis by using all variables in the univariate analysis.

## Data Availability

The data presented in this study are available on request from the corresponding author. The data are not publicly available due to ethical issue.
